# How interventions to maintain services during the COVID-19 pandemic strengthened systems for delivery of maternal and child health services: a case-study of Wakiso District, Uganda

**DOI:** 10.1080/16549716.2024.2314345

**Published:** 2024-02-21

**Authors:** Steven Ndugwa Kabwama, Rhoda K. Wanyenze, Neda Razaz, John M. Ssenkusu, Tobias Alfvén, Helena Lindgren

**Affiliations:** a Department of Global Public Health, Karolinska Institutet, Stockholm, Sweden; b Department of Community Health and Behavioral Sciences, Makerere University School of Public Health, Kampala, Uganda; c Department of Disease Control and Environmental Health, Makerere University School of Public Health, Kampala, Uganda; d Department of Medicine, Karolinska Institutet, Stockholm, Sweden; e Department of Epidemiology and Biostatistics, Makerere University School of Public Health, Kampala, Uganda; f Paediatric Public Health Department, Sachs’ Children and Youth Hospital, Stockholm, Sweden; g Department of Women’s and Children’s Health, Karolinska Institutet, Stockholm, Sweden; h Department of Health Promotion, Sophiahemmet University, Stockholm, Sweden

**Keywords:** COVID-19, emergencies, service delivery, child health, maternal health, health system

## Abstract

**Background:**

Health systems are resilient if they absorb, adapt, and transform in response to shocks. Although absorptive and adaptive capacities have been demonstrated during the COVID-19 response, little has been documented about their transformability and strengthened service delivery systems. We aimed to describe improvements in maternal and child health service delivery as a result of investments during the COVID-19 response.

**Methods:**

This was a descriptive case study conducted in Wakiso District in central Uganda. It included 21 nurses and midwives as key informants and 32 mothers in three focus group discussions. Data were collected using an interview guide following the Systems Engineering Initiative for Patient Safety theoretical framework for service delivery.

**Results:**

Maternal and child health service delivery during the pandemic involved service provision without changes, service delivery with temporary changes and outcomes, and service delivery that resulted into sustained changes and outcomes. Temporary changes included patient schedule adjustments, community service delivery and negative outcomes such as increased workload and stigma against health workers. Sustained changes that strengthened service delivery included new infrastructure and supplies such as ambulances and equipment, new roles involving infection prevention and control, increased role of community health workers and outcomes such as improved workplace safety and teamwork.

**Conclusions:**

In spite of the negative impact the COVID-19 pandemic had on health systems, it created the impetus to invest in system improvements. Investments such as new facility infrastructure and emergency medical services were leveraged to improve maternal and child health services delivery. The inter-departmental collaboration during the response to the COVID-19 pandemic resulted into an improved intra-hospital environment for other service delivery. However, there is a need to evaluate lessons beyond health facilities and whether these learnings are deliberately integrated into service delivery. Future responses should also address the psychological and physical impacts suffered by health workers to maintain service delivery.

## Background

Between 2000 and 2019, progress in improving health service coverage increased the global life expectancy at birth from 66.8 to 73.3 years and healthy life expectancy rose by 20.8% in low-income countries [1]. However, the 2022 Sustainable Development Goals Report [2] noted that the COVID-19 pandemic had decelerated progress in achieving global health goals through reducing global life expectancy, halting progress towards Universal Health Coverage and disrupting access to essential health services. Essential health services such as maternal and child health were affected through disrupting supply chains for essential commodities like contraceptives and vaccines; transport challenges for pregnant women [3] and halting or postponing essential services like antenatal care and immunization due to fear of COVID-19 infections in health facilities [3,4]. These challenges presented a significant test of the resilience of already resource-constrained health systems particularly in low- and middle-income countries. Health system resilience is a multi-dimensional concept that includes a system’s capacity to absorb, adapt, transform and improve during and after a crisis [5,6]. Absorptive capacity refers to a health system’s ability to maintain the quantity and quality of health services equitably during a health crisis. Adaptive capacity refers to its ability to maintain service delivery with fewer or different resources, whereas transformative capacity refers to the ability to adjust functions and structure when responding to a crisis. The absorptive and adaptive capacities of health systems during the COVID-19 pandemic have been extensively documented. Findings across countries noted that national health systems exhibited adaptive and absorptive capacities through the use of telemedicine to replace in-person consultations, community outreaches to provide information on service changes, introduction of new modalities for dispensing medicines [7], introduction of mobile medical teams and redirection of patients to other health facilities [8]. In Uganda, there was establishment of coordination structures for essential health services maintenance, introduction of self-management, leveraging the community health workforce, as well as patient networks to distribute medicines [7]. However, little has been documented about the short- and long-term transformability of health systems because of the COVID-19 pandemic – or the extent to which the pandemic served as a window of opportunity for novelty, creativity and innovation [9] resulting into improved service delivery and response systems. Loveday and colleagues [10] reported about how to leverage COVID-19 interventions to improve health system performance but did not assess whether their recommendations improved service delivery. In fact, the protracted nature of the pandemic has limited the implementation of systematic assessments of the COVID-19 response to identify successes and improvements as prescribed by the International Health Regulations (IHR) Monitoring and Evaluation Framework [11]. This information is relevant for health system recovery and strengthening. For countries to reassume the global health and development trajectories that had been recorded prior to the COVID-19 pandemic, it is important to evaluate and identify strategies and interventions that can be scaled up to improve health system performance. This study that aimed to identify the absorptive and adaptive strategies and interventions implemented to maintain the delivery of maternal and child health services and which had been adopted to transform and improver routine service delivery.

## Methods

### Study area

The study was conducted in Wakiso District, Uganda, in East Africa. The district is located in central Uganda and has a population of 3.1 million people which is the highest district population in the country [12]. The district was chosen because it registered the highest number of COVID-19 cases in the early months of the pandemic in Uganda [13] which enhanced the transferability of study findings to similar contexts [14].

### Study design

We used qualitative inquiry to conduct a descriptive case study [14,15] in which we investigated Wakiso District as a bounded system where the COVID-19 pandemic disrupted health service delivery. This approach aimed to build a detailed and nuanced understanding of the strategies the health system employed to sustain service delivery at the health facility level, and which had been adopted to improve routine delivery of maternal and child health services.

### Study participants

To improve the credibility and trustworthiness [14,16] of our study, we used a purposive sampling strategy to select 21 key informants that were directly engaged in the delivery of maternal and child health services at health facilities. These included nurses and midwives at the highest volume public and private health facilities in Wakiso District. The assistant district health officer for maternal and child health and the district biostatistician supported the selection of facilities. We selected key informants from public and private facilities at the level of Health Center IIIs, IVs and Hospitals as this is where the services that cover the continuum of care for maternal, neonatal and child health [17] in its entirety can be accessed. More details about each key informant have been provided as supplementary material (Supplementary Material 1).

In addition, the confirmability of the research [14] was enhanced by conducting focus group discussions (FGD) with 32 mothers who sought services at the health facilities in the district to corroborate findings from key informant interviews. FGD one had 10 participants, FGD two had 11 participants and FGD three had 11 participants. Mothers were eligible to participate in the FGD if they had at least 2 children both of whom were less than 3 years old. The rationale for this criterion was to obtain the service user perspectives from mothers who had received services during and after the peak of the pandemic.

## Data collection

**Figure 1.**
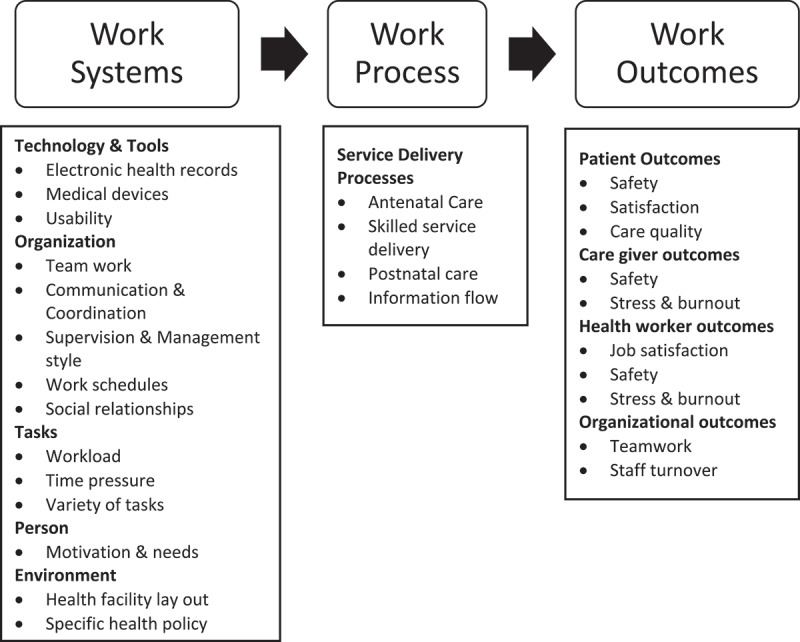
Systems Engineering Initiative for Patient Safety (SEIPS).

Data were collected using face-to-face interviews with key informants using a pre-tested interview guide with open-ended questions and probes. The interview guide was developed following the Systems Engineering Initiative for Patient Safety [18] which combines the work system model [19] and Donabenian’s quality of health care model [20] and covers both the demand and supply side of health care provision. The model can be used to identify organizational factors related to resilience such as coordination and team work as well as flexibility, adaptability and transformational resilience factors such as health infrastructure, technology and tools. Previous researchers have used the framework to evaluate the response to the COVID-19 pandemic [21], its impact on health service continuity [22] and the prevention of intra-hospital COVID-19 infections [23]. The framework has three main components including work systems (health care personnel, roles, tools & technology, internal and external environment), work processes (physical processes, e.g. antenatal care) and work outcomes (health worker, patient and caregiver outcomes – safety, stress and burnout, turnover) (Figure 1).

The first author and a female research officer with experience in conducting qualitative interviews conducted the interviews and FGDs in English and/or Luganda (local language spoken in Central Uganda). The interview guide provided an overall structure of the interview but allowed for additional probes and clarifications to improve the richness of the information gathered. All participants provided verbal consent to audio record the interviews which were later transcribed verbatim. Interviews lasted an average of 35 minutes. The shortest interview was 19 minutes, and the longest interview was 55 minutes. To improve the dependability of study findings [14], regular meetings were held to address challenges with scheduling interviews with key informants, refocusing of probes and clarifications to ensure study objectives are met, and the rate at which new information was being generated. During the interviews, data saturation started by the eighteenth interview and by the end of the twenty-first interview, no new information had been generated. The interviews and focus group discussions were conducted between October 2022 and February 2023.

## Data management and analysis

The first author listened to all the audio recordings and read the interview transcripts several times. The first author then manually generated a codebook based on themes in the interview guide. After coding, deductive content analysis [24] was conducted to generate sub-themes using the components of the SIEPS framework as a guide. We present quotes after a careful selection of statements that illustrate ideas, illuminate experiences and add to the overall appreciation of the study findings [25].

## Ethical considerations

The study protocol was approved by the Higher Degrees Research and Ethics Committee of Makerere University School of Public Health (Protocol #903) and registered with the Uganda National Council for Science and Technology (UNCST) (Approval #HS1121ES). Permission to conduct the study was also provided by the District Health Office of Wakiso District Local Government. Each study participant provided both oral and written informed consent to participate in the study.

## Results

Our findings revealed several broad themes whereby maternal and child health service continuity during the COVID-19 pandemic involved sustaining the routine delivery as well as adaptations and innovations to routine service delivery as shown in Table 1. Key informants noted that on the one hand, services such as antenatal care, facility deliveries, facility-based vaccination of children were delivered without altering modes of delivery and that services were delivered in the same way as before the pandemic.

**Table 1.** Themes of the adaptations and improvements for delivery of maternal and child health services.

Category	Temporary and short-term	Permanent and Sustained
Work Systems	Schedule changes	New Infrastructure, equipment & supplies
		New roles & assignments such as infection prevention and control committee members
		New information technology systems for data and training
Work Processes	Community service delivery	Integration of services such as ANC and family planning with immunization
		Multi-month commodity dispensing
		Increased role of community health workforce to include distribution of drugs and family planning commodities
Work outcomes	Increased work load	Workplace safety through improved infection prevention and control
	Stigma against health workers	Team work

**Figure 2.**
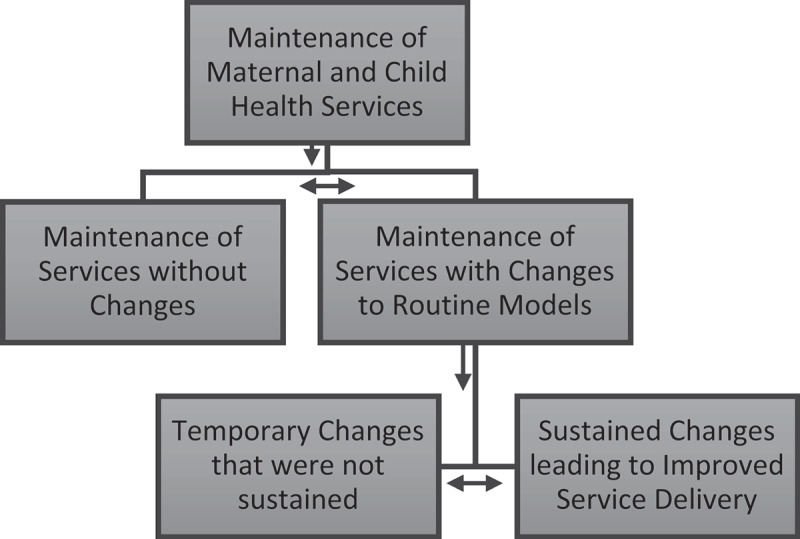
Broad themes from the key informant interviews and focus group discussions.

On the other hand, maternal and child health service delivery particularly in communities was maintained through altering existing modes of delivery. The adaptations and innovations for the maintenance of maternal and child health services across the domains of the Systems Engineering Initiative for Patient Safety framework can also be grouped further into two broad themes, i.e. those that were temporary and those that were sustained and improved service delivery (Figure 2). The sections below show the innovations and adaptations that were either temporary or sustained up to the time of the interviews, with quotes that illustrate the participants’ perceptions.

### Temporary and short-term changes

#### Schedule changes

Key informants described adjustments in work schedules to maintain service delivery because of the movement restrictions and to minimise the risk of infection by adhering to distancing measures within health facilities. They noted that prior to the pandemic, nurses and midwives worked in 5-day 12-hour shifts. However, during the pandemic, key informants delivered services in shifts of 72, 96 or 120 hours.

… the work hours were elongated because we were limited to movements so you had to come work for longer hours in that your colleague also gets to go and see people at home. Since the movements were limited we had to work like for more than 24hrs and more. Numbers of covid have gone down now the work schedules is normal. KII six, public health facility

Although this served to promote social distancing and prevent intra-hospital infections, it was not sustained because of the significant toll the schedules had on the social, physical and mental well-being of the health workers.

#### Community service delivery

Health workers also maintained service delivery by moving services from health facilities to communities. According to the participants, this was because of the movement restrictions as well as the anxiety and fear of COVID-19 infection in health facilities by patients. Community service delivery involved provision of family planning commodities, provision of antenatal care, dispensing ARV drugs for pregnant mothers among others. The strategy was effective in reaching persons who could not reach health facilities. However, it became ineffective and could not be sustained due to the difficulty in providing care in its entirety (i.e. from triage, consultation, diagnostic investigation and prescription) within community settings. As noted below, if someone required a specific service like blood test for example, this could not be offered in a community setting.

… some services are within the facility. So, when you visit the patient, you may see that there are some tests that might be needed and you may not have moved with other things to help in testing; let’s say the lab equipment. So, it could have been better if the person came (to the health facility). KII eight public health facility

In addition, they noted that some family planning options like implants could not be provided in communities. The other challenge was the strain on the existing health workforce as there were few health workers to deliver the services in the community while also sustaining facility-based services.

#### Increased work load

In terms of outcomes for the health workers, maintaining service delivery during the pandemic involved increase in workload. This was because of the increase in the number of patients in lower-level health facilities due to the dedication of the regional referral hospital to management of COVID-19 cases. Health workers suffered from fatigue, stress, burnout and reduced time for family as illustrated by the quote below.

… as health workers, we were very fatigued. Because you could find that you had some tasks here but then you had again to go and help out a colleague. And also emergencies could come up and it was hard to manage them. KII seven, public health facility

However, this was also not sustained and the workload reverted to pre-pandemic levels when the COVID-19 cases reduced and RRHs reverted to general health service delivery.

#### Stigma for health workers

Another temporary outcome for health workers was COVID-19 related stigma. Key informants reported that they experienced stigma from their communities who thought the health workers would infect them with COVID-19. This outcome was also not sustained as it was at the earliest months of the pandemic when the anxiety and fear within communities was very high.

… they (family) used to tell us either don’t come back home you are going to bring the disease, in the community for instance where I get water they wouldn’t want you to touch their tap, they tell you that doctor will bring the disease … . KII eighteen, Public Health Facility

### Permanent and sustained changes

#### New infrastructure, equipment and supplies

Key informants noted that there were efforts to improve the health facility infrastructure at lower levels of the health system in the district. They noted that some facilities received temporary structures like tents to increase space while others constructed entire maternity wards and out-patient-department facilities to promote social distancing. Other health facilities received funds from the Ministry of Health to construct isolation units while others constructed laboratories to strengthen diagnostic capacity. In addition, participants reported that health workers received infection prevention and control commodities such as gloves, aprons, face shields as well as hand washing equipment. Furthermore, health facilities received disinfection equipment such as autoclaves. Also, several health facilities received ambulances for transporting pregnant mothers to the national referral hospital. Health workers continued to use this equipment and supplies in the delivery of maternal and child health services.

Because of the pandemic, they gave us furniture; they gave us tents, hand washing equipment … they gave us an ambulance. We got the ambulance because we could complain that mothers are (arriving late) and dieing then we got an ambulance. KII five, Public Health Facility

#### Integration of services

Another strategy used to maintain service delivery, and which became sustained was the integration of services. For example, key informants described that the integrated services such as antenatal care, family planning as well as immunization to minimize the number of times the mother returns to the health facility. This integration was both at health facility level and the regularly scheduled community outreaches.

#### New roles and assignments

The maintenance of service delivery during the COVID-19 pandemic also introduced new roles and assignments for health workers. For example, key informants noted that infection prevention and control committees were established to promote adherence to infection prevention and control within the health facility by ensuring that all health workers and patients wear face coverings, ensuring that all service delivery stations had hand disinfectants and ensuring that all hand washing stations had soap and water for both patients and health workers. Other key informants noted that they were given new roles and assignments as staff had to work across departments, i.e. ANC, maternity and immunization.

… we had an IPC team, so it became active after covid, it became active during covid now like those moments of hand hygiene … They make sure we have sanitizer everywhere that group, then those posters that people will hand wash they put around hand washing equipment. Then we have this sodium hypochlorite, they make sure chlorine is around for disinfection … . KII five, Public Health Facility

#### New information and technology systems for data and training

Several key informants noted that information and technology systems were installed to ease the processes of data entry and record keeping during the COVID-19 pandemic. Systems were installed to record early infant diagnosis data as well as ANC data, e.g. age, address, and location for mothers on ART. These improved service delivery by saving time as patient data are already in a system. In addition, data could easily be retrieved in case a mother forgot their ANC card. Furthermore, the systems facilitated follow-up of mothers since their contact information was recorded in the system. The adoption of technology was noted by several informants.

… before we used to write, let me say those papers they used to provide us, we write, we give to the records and the records takes it but now the records has to take it now and send it via the phone and the records has to make sure that this report has to reach the ministry of health. KII three, Private Health Facility

#### Multi-month commodity dispensing

Dispensing drugs and family planning commodities for longer periods was also adopted. This strategy reduced the number of trips an individual had to make to the health facility and was especially effective for dispensing HIV medicines. Although the strategy started before the pandemic particularly for dispensing HIV medicines, it was scaled up during the pandemic for both HIV and non-HIV care, and was used even when the number of COVID-19 cases dropped.

… we could give them high supplies of the medicine like for three months or six months for those who adhere well so that they don’t keep coming. KII ten, Public Health Facility

#### Increasing role of community health workforce

Service continuity involved strategies that utilized the community health workers to distribute and deliver care in community settings. For example, community health workers identified pregnant mothers and encouraged them to seek care at the health facility. In addition, community health workers distributed family planning commodities, ARVs and antimalarials to pregnant mothers in communities. This increasing role of the community health workers was noted by several informants.

… before covid, we used not to distribute medicine using the village health teams… but, when covid came, we had to write their names down and go distributing medicine in their homes. We had to tell the members of the village health teams, that if so and so reaches this month of pregnancy, you are supposed to give them this medicine … . KII fifteen, Public Health Facility

The increasing role of the community health workers was corroborated by mothers who participated in the focus group discussions.

When it came to the time for giving birth, I was referred by the Village Health Team member and I was given free services. After giving birth, a contraceptive was brought home and they implanted it. Mother, FGD Two

#### Workplace safety

As a result of the COVID-19 pandemic and the interventions instituted to maintain service delivery, there were improvements in patient and health worker outcomes such as workplace safety. Key informants noted improvements in the implementation of infection prevention and control practices such as hand washing, wearing gloves and masking. These practices also improved infection prevention and control of the spread of other conditions like TB. The improvement in workplace safety is illustrated by the quote below:

… it has improved and the patients themselves know that they have to come to the facility when they are wearing a mask which reduces on infections because before we thought that it reduces on COVID alone but it had helped us prevent diseases like tuberculosis and coughs to see that it doesn’t spread amongst people and patients. KII eleven, Public Health Facility

#### Team work

Key informants noted better team work for the delivery of maternal and child health services. They described that during the COVID-19 pandemic, there was a need for staff to work across departments because of the increased work load. Roles were reassigned and redistributed to ensure uninterrupted delivery of services. The inter-departmental collaboration resulted into better teamwork and some key informants also noted improved individual capacity to deliver maternal and child health services because of having worked across departments.

We had to decide that we had to do this and we had to work together as a team to support each other. If someone was working at a point and they felt that they were overwhelmed, they could come and ask for assistance from even our department for someone to go and assist them. KII sixteen, public health facility.

## Discussion

This study aimed to identify strategies and interventions implemented to maintain the delivery of maternal and child health services and which had been adopted for routine service delivery. We found that on the one hand, key informants did not alter models of delivering maternal and child health services. On the other hand, systems for delivery of maternal and child health services were improved through investments in infrastructure, equipment and health information systems. The COVID-19 pandemic created the impetus for investments in infrastructure through construction of isolation units, extra outpatient and in-patient wards in order to increase space and limit disease transmission. In addition, emergency medical services were strengthened through procurement of ambulances. Prior to the COVID-19 pandemic, most countries in sub-Saharan Africa invested little to build robust systems for delivery of emergency services [26]. In fact, the Uganda Ministry of Health has previously highlighted the poor capacity of emergency medical services as a key challenge to health service delivery [27] and yet more than half of all ambulance complaints, particularly in rural areas, being maternal health–related emergencies [28]. The COVID-19 pandemic created the need for early identification, transport and management of cases to control spread and minimize transmission which prompted the investment in and improvement of emergency medical services. These investments were leveraged to improve delivery of emergency maternal and child health services. In addition, our study found that investments in information systems and technology infrastructure for managing COVID-19 related data also improved data systems for maternal and child health services. Other researchers have also highlighted the importance of leveraging data and information systems for COVID-19 to improve TB service delivery [10]. This will strengthen systems for monitoring the delivery and coverage of services and inform resource allocation for improvements.

Our study also found that inter-departmental collaboration and reallocation of roles and assignments during the COVID-19 response resulted in service delivery outcomes such as team work and workplace safety. Poor team work in the delivery of maternal and child health services increases the risks of maternal mortality and morbidity, perinatal mortality and morbidity, poor maternity perception of the quality of care as well as complaints and litigation by patients [29]. Effective teamwork results in employee cohesion, improves efficiency and reduces burnout among health care workers [30]. Maternal and child health service delivery involves multi-disciplinary teams in which communication and team work are critical to the safety and wellbeing of both patients and health care workers [31,32]. These improvements can be consolidated through teamwork trainings where the importance of teamwork and teamwork skills are presented, followed by the reinforcement of behaviours and attitudes [31] during delivery of maternal and child health services.

Another successful approach was the integration of health services during delivery. Services such as antenatal care were integrated with family planning and immunization services for both static posts and outreach services. The integration of health services has been described as a complex process without a definitive prescription of strategies to integration that will yield successful health outcomes [33,34]. However, several principles have been proposed including putting the patient at the centre of integration efforts and that services are accessible across the continuum of care [33]. Previous studies have shown that immunization and family planning services can be successfully integrated [35,36] while others have shown that antenatal care can be integrated with family planning [37]. Health system recovery efforts would benefit from evaluations of the impacts of integrating the three groups of services on patient and provider outcomes.

The study also found that certain interventions and outcomes were limited to emergency settings. These include work shift rescheduling, community service delivery and health worker outcomes such as increased workload and stigma. The increased workload is explained by the failure of rescheduling work shifts as has been reported elsewhere [38]. Responses to future public health emergencies should be cognizant of the physical and psychological impact that health workers endure to maintain service delivery during emergency responses. In addition, community service delivery had challenges such as stigma and difficulty in ensuring both vertical (care across a continuum) and horizontal (service availability) continuity of care. Responses to public health emergencies should address the challenges in order to use the strategies for service maintenance during emergencies.

### Strengths and limitations

The results of our study should be interpreted in light of some strengths and limitations. Our study employed the Systems Engineering Initiative for Patient Safety (SIEPS) model [18] as a guiding theoretical framework. Hodgines et al. have recently published a COVID-19 System Shock Framework [39] for assessing health system innovations during the COVID-19 pandemic. Although the framework provides a comprehensive blueprint for assessing health innovations during the COVID-19 pandemic, it contains elements such as financing, and governance and policy issues which would have been beyond the operational level of the health system where our study was situated. In addition, the SIEPS framework provided a blueprint for identifying the transformation of the health system through single-loop learnings based on actions at individual, team and organizational levels according to the WHO Flagship Report on Learning Health Systems [40]. Future research would benefit from evaluation of double-loop learnings which assess whether service delivery changes were based on clear models and assumptions and triple-loop learnings which assess whether efforts to document lessons are integrated in service delivery [40]. Another strength of the study was the detailed description of the findings with illustrative quotes which enhanced the authenticity of the study [14]. Another limitation of the study was the fact that one coder was involved in the generation of codes and themes from the interviews. However, the last author provided technical review, guidance and feedback about the codes and themes that were generated from the interviews.

## Conclusions

Interventions to maintain service delivery resulted into temporary and/or sustained changes and outcomes. Future responses to emergencies should be cognizant of the inevitable work system and process changes required to maintain service delivery and address the impacts of these changes on health workers. In spite of the negative impact the COVID-19 pandemic had on health systems, it created a concomitant impetus to improve health system capacity. Investments that improved work systems and processes such as new facility infrastructure, improving data systems and emergency medical services are being leveraged to improve the delivery of maternal and child health services. At operational level, the inter-departmental collaboration during the response to the COVID-19 pandemic resulted into an improved intra-hospital environment for delivery of other services. However, there is a need to evaluate learnings beyond the operational level of the health system and assessing whether efforts to document learnings are integrated into service delivery.

## Supplementary Material

Supplemental Material

## Data Availability

The dataset used for analysis can be availed upon reasonable request by writing an email to the corresponding author.

## Paper context

The COVID-19 pandemic had a negative impact on health systems globally. Little is documented about lessons from the response that informed recovery. This paper reports that the COVID-19 response in Uganda was associated with improvements in the delivery of maternal and child health services such as improved infrastructure and new service delivery strategies. This work contributes to health system resilience research and how this can be integrated into public health emergency preparedness and response efforts.
