# Supplementary material for: How interventions to maintain services during the COVID-19 pandemic strengthened systems for delivery of maternal and child health services: a case-study of Wakiso District, Uganda
Source: Glob Health Action. 2024 Feb 21;17(1):2314345. doi: 10.1080/16549716.2024.2314345 (PMC10883101; doi:10.1080/16549716.2024.2314345)
Supplement: Supplemental Material [file ZGHA_A_2314345_SM9608.docx]

**Supplementary Material 1. Details of Key Informants who Participated in the Study**

| **#** | **Health Facility** | **Public/ Private** | **Age** | **Occupation** |
| --- | --- | --- | --- | --- |
|  | HC IV | Public | 30-39 | Midwife and Focal Person, Elimination of Mother to Child Transmission (HIV/AIDS) |
|  | HC IV | Public | 30-39 | Midwife |
|  | HC III | Public | 20-29 | Midwife and In-charge, maternal and child health |
|  | HC IV | Public | 20-29 | Midwife and In-charge, maternal and child health |
|  | HC IV | Public | 40-49 | Midwife and In-charge, maternal and child health |
|  | HC IV | Public | 40-49 | Midwife and In-charge, maternal and child health |
|  | HC III | Public | 20-29 | Midwife |
|  | HC IV | Public | 40-49 | Midwife and In-charge, maternal and child health |
|  | HC III | Public | 20-29 | Midwife and Focal Person, Elimination of Mother to Child Transmission (HIV/AIDS) |
|  | HC III | Public | 20-29 | Midwife |
|  | Hospital | Private for Profit | 40-49 | Midwife and In-charge, maternal and child health |
|  | Hospital | Private for Profit | 30-39 | Midwife and In-charge, maternal and child health |
|  | HC III | Public | 40-49 | Midwife and In-charge, maternal and child health |
|  | HC III | Public | 20-29 | Midwife |
|  | HC IV | Private for Profit | 20-29 | Midwife |
|  | HC III | Public | 30-39 | Midwife |
|  | HC IV | Private for Profit | 30-39 | Nurse |
|  | HC IV | Public | 40-49 | Midwife |
|  | HC III | Public | 40-49 | Midwife |
|  | HC III | Public | 50-59 | Midwife |
|  | HC IV | Public | 20-29 | Midwife |
